# Using national data to model the New Zealand radiation oncology workforce

**DOI:** 10.1111/1754-9485.13448

**Published:** 2022-06-29

**Authors:** Alex Dunn, Shaun Costello, Fiona Imlach, Emmanuel Jo, Jason Gurney, Rose Simpson, Diana Sarfati

**Affiliations:** ^1^ Te Aho o Te Kahu/Cancer Control Agency Wellington New Zealand; ^2^ Southern District Health Board Dunedin New Zealand; ^3^ Health Workforce Directorate, Ministry of Health Wellington New Zealand; ^4^ Department of Medicine, School of Medicine University of Auckland Auckland New Zealand; ^5^ Cancer and Chronic Conditions (C3) Research Group, Department of Public Health University of Otago Wellington New Zealand

**Keywords:** Health Workforce, Health Workforce Modelling, Health Workforce Supply and Demand Planning, radiation oncology, radiotherapy/radiation therapy

## Abstract

**Introduction:**

Demand for radiation therapy is expected to increase over time. In Aotearoa/New Zealand, the radiation oncology workforce experiences high numbers of clinical hours but an intervention rate that is lower than in comparable countries, suggesting unmet treatment need. Accurate models on the supply and demand for radiation oncologists (ROs) are needed to ensure adequate staffing levels.

**Methods:**

We developed a demand model that predicted the future number of ROs required, using national data from the Radiation Oncology Collection (ROC) and a survey of ROs. Radiation therapy intervention and retreatment rates (IR/RTRs), and benign and non‐cancer conditions being treated, were derived from the ROC and applied to Census population projections. Survey data provided definitions of treatment by complexity, time spent in different activities and time available for work. Results were linked to radiation oncology workforce forecasts from a supply model developed by the Ministry of Health.

**Results:**

The demand model showed that 85 ROs would be needed in 2031, if current IR/RTRs were maintained, an increase from 68 in 2021. The supply model predicted a decrease in ROs over time, leaving a significant shortfall. Model parameters could be modified to assess the impact of workforce or practice changes; more ROs would be needed if average working hours reduced or IR/RTRs increased.

**Conclusion:**

Workforce models based on robust data collections are an important tool for workforce planning. The RO demand model presented here combines detailed information on treatment and work activities to provide credible estimates that can be used to inform actions on training, recruitment and retention.

## Introduction

In Aotearoa/New Zealand (hereafter NZ), around 25,000 people are diagnosed with cancer each year, a number that is expected to increase as the population ages.^1^ Cancer survival rates are improving for most cancers^1^ due to a range of factors, including advances in cancer treatment. One of the mainstays of cancer treatment is radiation therapy, which treats cancer with ionising radiation and requires a highly specialised workforce including radiation oncologists, radiation therapists and medical physicists. Internationally, workforce capacity is a major challenge in radiation therapy service delivery.^2^ Adequate staffing of radiation therapy centres is a key factor for achieving optimal cancer care,^3^ and in turn ensuring that the key treatment and outcome‐related goals of New Zealand's 2019–2029 Cancer Action Plan^4^ are met.

The radiation oncology workforce in NZ is small (60 practising ROs with 22 in training in 2018^5^; 68 practising ROs in 2020^6^). Workloads in terms of clinical hours are high, at a median of 50 h a week, compared to 41 h in Australia^7^ (and higher than the average for all doctors in NZ^6^), and symptoms of burnout are common.^8^, ^9^, ^10^, ^11^ Despite high workloads, the proportion of people with cancer who receive at least one course of radiation therapy (the intervention rate, or IR) is decreasing (from 35% in 2014 to 32% in 2018^12^) and is less than what is achieved internationally (37–52%).^13^, ^14^, ^15^ These low and declining rates may suggest a level of unmet need.

To understand how best to meet this need, we need to know how many ROs are required for current and future service delivery. International guidance on the optimal level of RO staffing varies, depending on differences in cancer incidence and socio‐economic factors in the population, adoption of technology within services and the distribution of professional roles and responsibilities.^16^ For example, a review of European guidelines on RO staffing levels found 27 recommendations from different countries ranging from 130–300 patients per year per RO.^17^ Recommendations are based on models that forecast future RO workforce requirements. A key consideration for these models is how to account for new and more complex treatment techniques that disproportionately increase workload. For these reasons, workforce models cannot be solely based on population estimates or cancer incidence, historic levels of staffing or funding, or number of treatments delivered^18^, ^19^ and need to be reviewed frequently to keep up to date with treatment changes.^20^ Models also need to factor in essential non‐clinical activities carried out in radiation oncology services that are not directly linked to patient care.^19^, ^21^

Robust workforce models are needed for workforce planning to ensure sufficient investment is made in training, recruitment and retention efforts to meet future demand for services. The first RO workforce modelling in NZ was published in 2012 by the Royal Australian and New Zealand College of Radiologists (RANZCR), Faculty of Radiation Oncology.^22^ This used RANZCR membership and workforce data, supplemented with survey results, to model RO supply and inputs from an Australian model to predict demand.^23^ The model predicted that 96 ROs would be needed to meet demand in 2022, for an IR of 45.2%, which was significantly higher than the existing IR. The RANZCR updated the model in 2016, with projections to 2026, but results were not published. However, a criticism of the methodology of this model is that it was based around a fixed estimate of the association between RO numbers and hardware, that is two ROs for every linear accelerator (LINAC). This approach may be considered overly simplistic, not responsive to changing roles of the workforce, and maladaptive to adjustment for complexity of treatment.

A different approach was taken in workforce modelling published as part of the National Radiation Oncology Linear Accelerator and Workforce Plan in 2014.^24^ This used an estimate of RO capacity of one RO per 214 patients per year, which was the national average at the time, assuming one RO equated to one full‐time equivalent (FTE) working a 40‐h week,^24^ even though the average weekly hours for ROs was 49 h in 2012.^25^ This model indicated that an increase in ROs would be needed if the IR increased to or beyond 45% or if the patient load was reduced to 175–200 courses per RO per year (from the actual average value of 213 per RO per year). This model also did not consider complexity of treatment.

To overcome the shortcomings of previous models, an alternative model was developed in 2019, using data from the Radiation Oncology Collection (ROC), a national, centralised database that contains comprehensive information on radiation treatment delivery, including type and number of treatments. The ROC data, alongside survey data on the real‐life work experiences of ROs, was used to develop a workforce demand model that could account for different complexities of treatment, non‐clinical and clinical activities of ROs, and actual hours worked rather than FTE estimates. Results from this new model can be compared to RO supply modelling undertaken by the Ministry of Health,^26^ which predicts the number of practising ROs in future years, based on current work patterns.

In this paper, we outline the content and development of the RO workforce demand model, present estimates of RO requirements based on a variety of scenarios and discuss how the model results have been used to inform RO workforce planning in NZ.

## Methods

### Default demand model

The demand model was developed in 2019 in consultation with ROs from the national Radiation Oncology Working Group (ROWG), a clinical advisory group to the Cancer Control Agency, Te Aho o Te Kahu. ROWG includes ROs from every radiation oncology centre in NZ (public and private) and has oversight of the ROC and activities related to service improvement. Data from the ROC were made public in 2018 (including data back to 2014) and provided, for the first time, comprehensive estimates of current and past intervention and retreatment rates (the proportion of all courses that are retreatments, RTRs), data on the number of benign and non‐cancer conditions being treated, and sufficient detail to allow treatments to be categorised according to complexity.

This provided a solid foundation for developing a new RO workforce model that could update, refine and validate previous workforce estimates from the RANZCR (Table 1 summarises all the data inputs into the demand model). The IR and RTR estimates were applied to numbers of cancers from the New Zealand Cancer Registry^27^ projected into the future based on static (3 year average) incidence rates applied to Census‐based population projections,^28^ to predict the future expected number of treatment courses.

**Table 1:** Demand model inputs

Input	Data source(s)	Value in 2021	Value in 2031
Projected new cancer registrations by year	Cancer Registry (Ministry of Health), for cancer registrations (2017–2019 average incidence; Statistics New Zealand District Health Board (DHB)† population projections Statistics New Zealand DHB Summary Projections 2013‐base (2019 Update)	27,725	34,291
Current radiation therapy intervention rate (IR) and retreatment rate (RTR)	Radiation Oncology Collection (ROC), average overall rates for NZ in 2017–18 (rates vary by DHB)	IR = 33.4%; RTR = 24%	As in 2021
Number of cancer treatment courses per year	Calculated from the data sources above (number of projected cancer registrations multiplied by the IR and RTR)‡	9260 first courses; 2924 retreatment courses	11,453 first courses; 3617 retreatment courses
Number of courses of radiation for benign/non‐cancer conditions	ROC, scaled to increase in proportion with population projections	1328 courses	1732 courses
Total number of treatment courses per year	From sources above – sum of cancer and benign/non‐cancer treatment courses	13,512 courses	16,802 courses
Proportion of courses by complexity of treatment	ROC captures treatment type/technique, classified as: Low complexity (simple fields) Medium complexity (3D conformal radiation therapy) High complexity (IMRT/VMAT/SABR) Very high complexity (stereotactic radiosurgery, brachytherapy) Overall proportions for NZ from 2018 (proportions vary by DHB)	Low = 40%	As in 2021
		Medium = 34%	
		High = 25%	
		Very high = 2%	

Number of treatment courses per year by complexity	Total number of treatment courses per year multiplied by the treatment complexity proportions	Low = 5383	Low = 6693
		Medium = 4545	Medium = 5651
		High = 3326	High = 4135
		Very high = 259	Very high = 322
Time required for RO planning/prescribing/ treatment supervision according to complexity of treatment	Survey	Low = 1 h	As in 2021
		Medium = 1.5 h	
		High = 3 h	
		Very high = 7 h	
Time required for first specialist assessment (FSA)	Survey	90 min	As in 2021
Ratio of FSA to course (proportion of FSAs that result in the delivery of a course)	Survey	1.01	As in 2021
Time required for follow‐up	Survey	30 min	As in 2021
Number of follow‐ups per course	Survey	4.1	As in 2021
RO clinical hours per treatment course, by complexity	From sources above – sum of RO time for planning, FSA time multiplied by ratio of FSA to course, follow‐up time multiplied by number of follow‐ups, by complexity of treatment	Low = 4.6 h	As in 2021
		Medium = 5.1 h	
		High = 6.6 h	
		Very high = 10.6 h	
RO clinical hours per year by course complexity	From sources above – number of treatment courses per year by complexity multiplied by total RO hours by complexity	Low = 24,572 h	Low = 30,554 h
		Medium = 23,020 h	Medium = 28,623 h
		High = 21,833 h	High = 27,148 h
		Very high = 2739 h	Very high = 3406 h
Proportion of time spent on supporting clinical activities	Survey	40%	As in 2021
Total RO clinical hours per year	From sources above – sum of clinical hours per year by course complexity, including the additional 40% of time for supporting clinical activities (sum of hours divided by 0.6 [1–0.4])	Total clinical hours = 120,274	Total clinical hours = 149,551
Available working time per RO (for clinical and non‐clinical activities)	Survey	Weekly hours per RO = 55	As in 2021
		RO working weeks per year = 41	
Non‐clinical time per week per RO	Survey. Calculated as 24% of weekly hours to a maximum of 12 h	12 h per week§	As in 2021
Clinical time per week per RO	Weekly hours minus non‐clinical time	43 h per week	As in 2021
Total clinical time per year per RO	Clinical time per week multiplied by RO working weeks per year	1,763 h	As in 2021
Number of ROs required (per year)	Total RO clinical hours per year divided by total clinical time per year per RO	68	85

IMRT, intensity‐modulated radiotherapy; SABR, stereotactic ablative radiotherapy; VMAT, volumetric‐modulated arc therapy.

† DHBs are regions in NZ responsible for health service delivery to their regional population. Average cancer incidence is applied by age and major cancer type to DHB projections to give expected numbers of cancer, by cancer type, for each DHB; amalgamated into a national estimate. The additional granularity by DHB is used in LINAC demand modelling, not discussed in this paper.

‡ These numbers change with changes to the IR and RTR (e.g. using scenarios of ‘best current DHB rates’ or ‘international rates’). All the subsequent numbers in this table are based on the default current IR/RTR (33.4%/24%).

§ The maximum of 12 h is used in the model, as non‐clinical time is capped at 12 h in standard employment agreements and 24% of 55 h per week is 13.2 h. If weekly hours dropped below 50 h per week, this value would decrease.

The next stage of development was to undertake a survey of ROs, co‐ordinated through ROWG members, to estimate clinical and non‐clinical time commitments for ROs in NZ, including amount of time spent on planning and review according to treatment complexity (defined as low to very high complexity based on therapy type, see Table 1). Survey results were discussed at a ROWG meeting and national values for each workload item were chosen by consensus, usually an average or median across the centres. The national values are the default values used in this paper; values specific to DHBs and individual radiation therapy centres can also be applied (but are not presented here).

From the survey and ROWG discussion, the components of RO work were defined into three main areas:

- Direct clinical activities (including first specialist assessments [FSAs), follow‐up appointments and all aspects of treatment planning and supervision). FSA time includes dictation, data entry as well as patient time;
- Supporting clinical activities (includes time spent on call, in multidisciplinary meetings, weekend ward rounds, phone calls, triage, that is all clinical activities not included in the direct clinical activities);
- Non‐clinical activities (teaching, other meetings, research, audit, management, administration).

Survey results confirmed the proportion of time spent on these activities, the average amount of time spent per course of treatment by complexity, and the average amount of time ROs were available for work. All measures were of actual time/hours rather than FTE and were used to calculate total RO clinical hours, which could then be used to predict the number of ROs needed depending on the number of cancer registrations and IR/RTR. For available working time per RO (for clinical and non‐clinical activities), 55 h per week was selected. This was considered to reflect a balance between the average hours worked by NZ ROs as reported during the survey, and an agreed absolute maximum sustainable workload for ROs.

The model was built in Microsoft Excel 2016 (Microsoft Inc., Redmond, WA, USA) and refined after feedback from the sector. Results in this paper are from the Oct 2020 1.3 version of the model, with a base year of 2019 and projections starting from 2020.

### Variations to demand model assumptions

The model can be adjusted to show the impact on the number of ROs required given different scenarios, for example increased (or decreased) complexity of treatments, or changes in RO working hours. Of particular interest is the impact of an increased IR and RTR. The default model uses the national IR/RTR from 2017 to 2018 (IR = 33.4%; RTR = 24%), but these rates vary across District Health Boards (DHBs). We present results based on two alternatives: the ‘best current IR/RTR’, which are the highest rates achieved across any DHB in NZ (IR = 41.9%; RTR = 33%), and the ‘international IR/RTR’, which is set at rates or benchmarks commonly reported internationally (45%/35%).^14^, ^24^ We assume that these higher proportions indicate more timely, accessible and appropriate treatment, that individual DHB rates are achievable nationally, and that the default IR/RTR indicates a level of unmet need.

### Supply model

We used data from a supply model developed and maintained by the Ministry of Health, which uses information on medical practitioners sourced from the New Zealand Medical Council from registration and survey data collected at the renewal of annual practising certificates (APC). Registration data includes demographics, country of qualification, vocational scope of practice and registration dates; work history, including average working hours, were collected from the survey. The supply model forecasts how many ROs will be practising in future years, based on work patterns (exit, entry and re‐entry patterns) from the past three years and average working hours by age group.^26^, ^29^ The model assumes that these work patterns remain stable over time and that there are no changes in practice or models of care.

### Model validation

The demand model was tested against the RANZCR model (mentioned in the Introduction), and results were found to be highly similar. Numbers from the model were checked against actual staff numbers for several radiation therapy centres in NZ; for example, predictions were consistent with the actual number of ROs in one centre that was identified by ROWG as likely being understaffed and also for another centre identified as being likely to be adequately staffed. The model has been endorsed by the Faculty of Radiation Oncology (RANZCR).

## Results

### Demand model predictions with varying intervention and retreatment rates alongside forecast supply of radiation oncologists

**Fig. 1:**
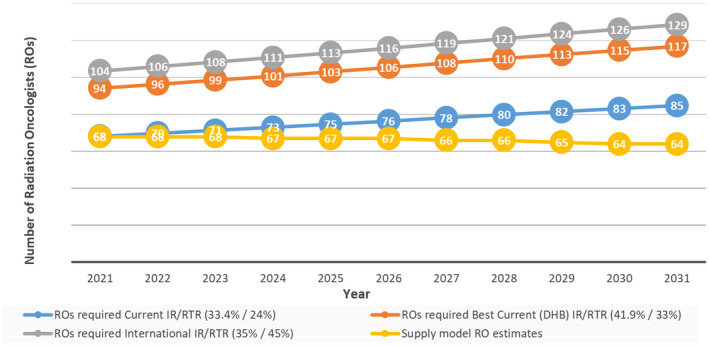
Radiation oncologists (ROs) required with different intervention rates (IR) and retreatment rates (RTR), with supply model estimates.

Figure 1 shows results from the demand model for 10 years (2021–2031), using the default values and based on current radiation therapy IR/RTR, the best DHB IR/RTR and the international IR/RTR.^14^ To maintain the current IR/RTR, NZ would need 68 ROs in 2021 and 85 ROs by 2031. If all DHBs achieved the highest rates recorded regionally in NZ, we would need 94 ROs in 2021 and 117 ROs in 2031. If NZ wanted to achieve an IR and RTRs comparable to Australia and elsewhere, we would need 104 ROs in 2021 and 129 ROs in 2031.

The results of the supply model reveal a significant shortfall of ROs, predicting that we will have fewer ROs in 2031 than today. The forecast numbers of ROs would be unable to meet demand for radiation therapy even if current IR/RTRs continued. Around a doubling of the RO workforce would be needed to meet demand with higher IR/RTRs.

### Demand model predictions based on other scenarios

Table 2 shows the impact on predictions from the demand model when values for individual parameters are changed for the outcome of required ROs in 2031 and for the three differing IR/RTR scenarios. For example, the proportion of time spent on supporting clinical activities was 40% in the default model. If this was reduced to 30%, then only 73 ROs would be required in 2031 to maintain the current IR/RTR (compared to 85 in the default model); but 102 ROs would be needed if 50% of time was spent on supporting clinical activities.

**Table 2:** Estimated number of Radiation Oncologists required in 2031 under a range of conditions

Model inputs	Number of Radiation Oncologists required in 2031			
	IR/RTR			
		Current 33.4%/24%	Best DHB 41.9%/33%	International 45%/35%
Default model results (all values at national defaults)		85	117	129
Model results when input values are changed (all other values set at default)				
	Default value			
Weekly hours worked per RO: 40 h	55 h	120	166	182
Proportion of time spent on supporting clinical activities: 30–50%	40%	73–102	100–140	110–154
Number of follow‐ups per course: 2–5.2	4.1	68–94	94–129	103–142
Duration of follow‐up: 20–45 min	30 min	74–101	102–139	112–153
Low complexity planning time: 0.5–2 h	1 h	82–91	113–126	124–138
Medium complexity planning time: 1–2 h	1.5 h	82–87	113–121	125–133
High complexity planning time: 2–4 h	3 h	81–89	112–122	123–135
Very high complexity planning time: 4–10 h	7 h	84–86	116–118	127–130
FSA duration: 60–95 min	90 min	77–86	106–119	116–131
FSA to course ratio: 0.82–1.16	1.01	80–88	111–122	122–134

The range of values tested in Table 2 derive from the range of results obtained from the survey of ROs. The parameters with the highest impact on the number of ROs required are weekly hours worked, proportion of time spent on supporting clinical activities and number and duration of follow‐up(s). At the most extreme, 120 ROs would be required in 2031 (under the current IR/RTR scenario) if ROs worked an average of 40 h per week.

Changes in planning time for courses of differing complexity have a more modest impact on the predicted number of ROs required – only a few more ROs were needed if more time was taken for planning for any one type of treatment complexity. However, if all of the upper values in planning time were applied to all levels of treatment complexity, the difference would be more pronounced (99 ROs required in 2031, under the current IR/RTR – data not shown in table).

The model can be also being used to estimate the impact of changes in practice, such as increased use of complex treatments (which will increase demand). For example, stereotactic ablative radiotherapy (SABR) may become the standard of care in the treatment of oligometastatic disease, where small tumours from the original primary cancer spread to other parts of the body, as recent trials show improved survival with this treatment.^30^, ^31^ In the public health system in NZ (excluding private cancer centres), the number of treatment courses for oligometastatic disease has increased by (on average) 74 per year since 2012, representing an additional 1.6 RO capacity (at 6.6 h per course, using default model values). If more of this type of treatment is required, the workforce will need additional capacity to cope with this increase in demand.

## Discussion

Previous RO workforce models have estimated demand for ROs using proxy measures such as number of doctors per 100,000 population^26^ or number of ROs required per LINAC.^22^ The model employed in this study uses real‐life data from the national ROC on types of treatment, supplemented with survey data, to create a comprehensive and validated demand analysis. The ROC was initially conceived as a quality improvement tool, but has had additional utility in workforce and LINAC capacity planning, as can be seen from its application in the current study.

Supply modelling for such a small workforce is subject to inaccuracies, since it only takes a few individuals to diverge from the model assumptions to have a significant impact (e.g. if more ROs decide to retire early, due to workload pressure; or more ROs leave NZ for better pay and working conditions overseas; or fewer internationally trained ROs are recruited). The supply model in the current study, using more granular, age‐specific work pattern data, improved on a previous model that did not adjust for age,^22^ since entry, exit and re‐entry rates vary considerably by age. Even so, the supply model forecasts for ROs have a larger margin of error than those for larger workforces, because of the small workforce size, but are still useful for predicting the direction and potential magnitude of change over time. In terms of the demand model, a potential limitation is the use of self‐reported estimates of time spent on planning and supporting clinical activities, which may overestimate these values. To mitigate this, we used average values in the default model and tested the impact of changing these values (Table 2), which showed that even with the lowest reported values, the shortfall in ROs persisted.

One advantage of the current model is the ability to adjust parameters to predict the impact of new policies, changes in practice and workload. If ROs are expected to spend more time on treatment planning, due to increased use of more complex therapies, less time is available to see new patients, and more ROs will be needed to maintain the current IR/RTR. The scenario of reduced working hours for ROs (to 40 h a week) is not unrealistic, given high rates of burnout, more ROs wanting to work part‐time or fewer hours^7^, ^32^ and an ageing workforce who may want to reduce hours when nearing retirement (the average age of ROs in 2020 was 51 years).^6^

The current model has been used by the Ministry of Health (Health Workforce) to prioritise radiation oncology as a vulnerable workforce,^33^ because of the magnitude of the shortage and the potential life‐threatening impact of serious shortages. Previous research has established that the number of RO trainees in NZ is insufficient to replace ROs who are expected to retire, let alone to accommodate increased preferences for part‐time work, provide protected time for research and mitigate unwanted overtime working, and to increase capacity in the face of increased demand and complexity of treatments.^5^, ^34^ This is consistent with international literature that describes large deficits in the existing radiation therapy workforce and infrastructure, with more predicted for the future.^2^, ^35^

### Addressing the deficit in RO capacity

Approaches to increasing the RO workforce include increased funding for RO training positions, retention of older ROs,^22^ retention of local RO fellows and attracting more international medical graduates. The health system in NZ already relies heavily on doctors who completed their training overseas, with more than 50% of ROs being international medical graduates in 2016.^36^ Average retention rates for New Zealand graduates five years post‐vocational registration are around 80% overall,^6^ but 42% of new ROs undertake a fellowship, mostly overseas due to limited availability in NZ, and not all of these ROs return.^5^ Many external factors influence retention and recruitment rates, including immigration, economic and labour market policies. Thus, attracting international graduates, and New Zealand graduates post‐fellowship, is challenging, especially as graduates are better paid overseas, have access to centres of research excellence and work fewer hours.^22^ ROs in NZ have little time for research, although clinical trials have benefits for patients.^5^

With demand for radiation therapy outstripping the supply of ROs, the workforce may need to be supported with interventions to reduce burnout.^37^, ^38^ Risk of burnout may be reduced by interventions focused on competence, relationships and autonomy at personal and institutional levels,^11^ co‐worker and supervisory support,^38^ and job satisfaction initiatives such as ongoing education, mentoring and management of workload and time demands.^8^, ^10^ Regular workforce surveys may identify where additional workplace supports are needed to alleviate stress and burnout and maintain a healthy and stable workforce in NZ. Workforce surveys can also provide a check to the validity of the workforce model; other checks could include wait times and access to radiation therapy (including access to more complex treatments).^39^

### Strengths and limitations of study

This study is among the first to use national‐level data to drive a RO demand model and provide credible estimates that can be used to inform actions on training, recruitment and retention. We note that the survey used for this study could be further refined in future to allow for more precise calculation of clinical activities, given the importance of these estimates within the RO demand model. This paper has not examined staffing shortages of radiation therapists and medical physicists, but these workforces are also understaffed.^40^ Shortages in these workforces also limit the ability to increase radiation therapy IRs, as does the number of LINACs, and addressing all of these shortages will be needed to improve access to radiation therapy services. Radiation therapists in advanced practice roles have the potential to increase capacity and patient volumes and throughput,^41^ but will require commitment to the development, implementation and evaluation of these roles.^42^ We also note that there have been recent advances in Artificial Intelligence (AI), which may, in the future, improve the quality and efficiency of radiation therapy delivery.^43^ While not directly included within the RO demand model, it could be incorporated once it becomes clear where in the clinical pathway this technology might save RO time in the New Zealand context, and the extent of these savings.

In conclusions, adequate staffing levels are needed to provide safe and high quality radiation therapy. Our models predict a large mismatch between the supply of radiation oncologists in NZ and demand for radiation therapy in the coming decade, with this mismatch even more pronounced if intervention rates and retreatment rates are to increase from their current rates. The demand model drew on robust data from a national collection that allowed complexity of treatment to be included, and survey data that provided important details on workload and capacity. Trusted workforce models that can be adjusted to account for changing practices and alternative scenarios are a useful tool for workforce planning and have been critical in NZ in making the radiation oncology workforce a priority for investment.

## Ethical approval

Ethics approval was not required for this project. The demand model used aggregate data from the Radiation Oncology Collection, with the oversight of the Radiation Oncology Working Group. Data for the supply model was obtained under the remit of the Health Practitioners Competence Assurance Act 2003, section 13A, that requires the Medical Council of New Zealand to provide the Director General of Health with workforce information and stipulates that no identifiable information will be published.

## Data Availability

Data for the demand model are publicly available from the Radiology Oncology Online Tool (https://minhealthnz.shinyapps.io/radiation‐oncology‐online‐tool‐test‐version‐2/). Data for the supply model are available from the Ministry of Health on request.
